# RURAL/URBAN DIFFERENCES IN LIFETIME OCCUPATION AND ALL-CAUSE MORTALITY AMONG MEXICAN ADULTS

**DOI:** 10.1093/geroni/igac059.2918

**Published:** 2022-12-20

**Authors:** Mariela Gutierrez, Rebeca Wong, Yong-Fang Kuo

**Affiliations:** University of Texas Medical Branch, School for Public and Population Health, Galveston, Texas, United States; University of Texas Medical Branch, Galveston, Texas, United States; University of Texas Medical Branch at Galveston, Galveston, Texas, United States

## Abstract

Mortality is affected by occupation dimensions, such as physical and mental demands, environmental exposures, and access to resources. Few studies have examined the relationship between occupation and mortality in countries with shifting occupation profiles, such as Mexico. We used 2001–2018 data from the Mexican Health and Aging Study to investigate the association between lifetime occupation type and all-cause mortality among participants aged 50 and older. We grouped participants’ longest-held occupations into five categories: (1) no main job; (2) agriculture; (3) domestic, service; (4) administrative, professionals, sales; and (5) production, industrial, transportation. We used Cox Proportional Hazard regression to study death risk associated with occupation, adjusting models for baseline demographic, health, and job characteristics. We stratified analysis by rural and urban to account for differences in occupation composition. Our sample included 10,482 participants, with 4,147 deaths occurring during follow-up. The percentage of participants in administrative, professional or sales was higher in urban areas than rural areas (60.7% vs. 20.5%). The percentage of participants in agriculture was higher in rural areas than urban areas (81.8% vs. 18.2%). Compared to professionals, participants in agriculture, industry, or transportation had a slightly higher risk of death (HR=1.65, 95% CI 1.59 to 1.77). Agriculture workers in rural areas had a higher risk of death than non-agriculture workers in rural areas. Considering rural/urban differences in occupations and their effect on mortality can inform policy aimed at improving the working environment for Mexican adults. Understanding rural/urban differences in mortality associated with occupation can elucidate socioeconomic inequalities in mortality.

